# Deletion of the Transcriptional Regulator MucR in *Brucella canis* Affects Stress Responses and Bacterial Virulence

**DOI:** 10.3389/fvets.2021.650942

**Published:** 2021-06-25

**Authors:** Jiali Sun, Hao Dong, Xiaowei Peng, Yufu Liu, Hui Jiang, Yu Feng, Qiaoling Li, Liangquan Zhu, Yuming Qin, Jiabo Ding

**Affiliations:** ^1^National Reference Laboratory for Animal Brucellosis, China Institute of Veterinary Drug Control, Beijing, China; ^2^Veterinary Diagnostic Laboratory, China Animal Disease Control Center, Beijing, China; ^3^Institute for Laboratory Animal Resources, National Institutes for Food and Drug Control, Beijing, China

**Keywords:** *Brucella canis*, MucR, virulence, RNA-Seq, stress responses

## Abstract

The transcriptional regulator MucR is related to normal growth, stress responses and *Brucella* virulence, and affects the expression of various virulence-related genes in smooth-type *Brucella* strains. However, the function of MucR in the rough-type *Brucella canis* remains unknown. In this study, we discovered that MucR protein was involved in resistance to heat stress, iron-limitation, and various antibiotics in *B*. *canis*. In addition, the expression level of various bacterial flagellum-related genes was altered in *mucR* mutant strain. Deletion of this transcriptional regulator in *B. canis* significantly affected *Brucella* virulence in RAW264.7 macrophage and mice infection model. To gain insight into the genetic basis for distinctive phenotypic properties exhibited by *mucR* mutant strain, RNA-seq was performed and the result showed that various genes involved in translation, ribosomal structure and biogenesis, signal transduction mechanisms, energy production, and conversion were significantly differently expressed in Δ*mucR* strain. Overall, these studies have not only discovered the phenotype of *mucR* mutant strain but also preliminarily uncovered the molecular mechanism between the transcriptional regulator MucR, stress response and bacterial virulence in *B*. *canis*.

## Introduction

In 1966, *Brucella canis* was first isolated from aborted tissues and vaginal discharge of beagles (1). In dogs, *B*. *canis* causes reproductive failure, while it causes fever, chills, malaise, peripheral lymphadenomegaly, and splenomegaly in humans (2). Recent studies have demonstrated that the intracellular trafficking route of *B*. *canis* was indistinguishable from that of *B. abortus* and a less robust response was observed in mice infected with *B*. *canis* compared with *B*. *abortus* in terms of proinflammatory cytokines, interferon-gamma levels, splenic inflammation, and hepatic granulomas (3). Another study indicated that only a dose of *B*. *canis* (up to 10^9^ CFU) could induce splenomegaly in infected mice at 1 and 2 weeks post-inoculation and the suitable challenge dose (1 ×10^7^ CFU) for investigating vaccine safety for *B*. *canis* seemed to be about 10^3^-fold higher than that of smooth-type *Brucella* strains (4). It seems that *B. canis* is less pathogenic than other smooth-type *Brucella* species in this murine model.

Due to the low prevalence of brucellosis caused by *B. canis*, which is less pathogenic than other *Brucella* species, most countries have not established clear protocols for its prevention and the public risk of this pathogen is usually ignored. Additionally, comparatively little is known about the pathogenic mechanism and virulence factors of *B*. *canis* strains.

*Brucella* strains do not produce classical virulence factors, such as cytolisins, exotoxin, exoenzymes, fimbria, plasmids, and drug-resistant forms. Lipopolysaccharide (LPS), T4SS secretion system, and BvrR/BvrS system have been considered to be major virulence factors allowing interaction with the host cell surface, the formation of an early, late BCV (*Brucella* Containing Vacuole) and interaction with the endoplasmic reticulum (ER) when the bacteria multiply (5). Besides, some transcription regulators have been proved to be involved in *Brucella* virulence, such as the well-studied LuxR-type regulator VjbR (6).

The virulence-related transcriptional regulator MucR, which is a member of the Ros/MucR family, is considered to control the expression of various genes involved in the successful interaction of α-proteobacteria with their eukaryotic hosts (7–9). In *B*. *melitensis* and *B*. *abortus*, the transcriptional regulator MucR is an extensively studied virulence factor and *mucR* mutant strain has been considered to be a promising vaccine candidate in mice model against both intraperitoneal and aerosol challenge (10). In *Brucella* strains, previous studies have also demonstrated that MucR protein is involved in resistance to environmental stresses, such as acidic stress, oxidative stress, detergent, cationic peptide, and iron deficiency (9, 11). Additionally, previous transcriptomic and microarray analyses have revealed that MucR could directly and indirectly affect the expression of hundreds of genes involved in metabolism, cell wall/envelope biogenesis, replication, and translation (9, 12). In recent studies, various target genes, which were directly regulated by MucR protein, were also identified, such as *babR, virB* genes, *bab1_0746, bab1_1035, bab1_1605*, and *bab1_1893* (12, 13). Compared to the well-studied MucR protein in smooth-type *Brucella* strains, no study has been carried out so far to demonstrate the function of MucR protein in *B*. *canis* strains.

Herein, we constructed *mucR* in-frame deleted mutant strain and complemented strain in *B*. *canis*. Then, we assessed the phenotypes of *mucR* mutant strain, such as growth characteristics and bacterial virulence in both macrophages and mice infection models. Moreover, the sensitivity of Δ*mucR* under various stress conditions was determined. Finally, RNA-seq analysis was performed to investigate the changes of the transcriptional profile of gene expression in *mucR* mutant strain compared with its parent strain RM6/66.

## Materials and Methods

### Construction of *mucR* Deletion Mutant Strain

Construction of the suicide plasmid was performed as previously reported (14). Specifically, for *B. canis* RM6/66 *mucR* gene mutant, a 433-bp upstream fragment and a 500-bp downstream fragment of *mucR* gene were amplified using D-mucR-UF/UR and D-mucR-DF/DR primers, respectively (Supplementary Table 1). The two fragments were then used as templates for the second round of overlap polymerase chain reaction (PCR). The purified PCR product was digested with *Kpn*I and *Hind*III restriction enzymes and cloned into the puc19-*sacB* plasmid, which was verified using PCR and sequencing.

The *B. canis* RM6/66 *mucR* gene unmarked in-frame deletion mutant was constructed with allelic replacement using a two-step method (14). The suicide plasmid was transformed into the wild-type *B. canis* RM6/66 strain by electro transformation. Single crossover integrates were then selected on tryptic soy agar (TSA) supplemented with ampicillin (100 μg/ml). The second crossover selection was conducted by plating the bacteria on TSA containing 5% sucrose. Ampicillin sensitive colonies were selected and verified by PCR and sequencing.

### Construction of *mucR* Complemented Strain

The *mucR* fragment, including the promoter sequence (500-bp of the intergenic region upstream of *mucR* start codon), was amplified with C-mucR-F/R primers (Supplementary Table 1). The PCR products were ligated to pMR11 plasmid, which was then referred to as the complementation plasmid pMR-*mucR*. To construct the complemented strain (C*mucR*), pMR-*mucR* was electroporated into *mucR* deletion mutant and then the cells were plated onto TSA containing ampicillin to detect the presence of pMR-*mucR* in the complemented strain. The resulting strains were verified by PCR and sequencing. For determination of minimum inhibitory concentrations (MICs) of antibiotics, the complemented strain (C*mucR*1) with chloramphenicol resistance was constructed using pBBR1MCS-1 plasmid using the same primers (C-mucR-F/R) as previously reported (15).

### RNA Isolation

For RNA-seq assay, *Brucella* strains (RM6/66 and Δ*mucR*) were grown in three different tubes with TSB at 37°C from a single colony until the log phase was reached. Total RNA was isolated using TRIzol according to the manufacturer's instructions. Residual DNA in the samples was removed using DNase I. RNA concentration and purity were determined spectrophotometrically using an ND 1000 spectrophotometer (Thermo Scientific, Wilmington, USA).

### RNA-Sequencing

The sequencing library of each RNA sample was prepared using NEB Next Ultra Directional RNA Library Prep Kit for Illumina as recommended by the manufacturer. Briefly, RNA fragments were reverse-transcribed and amplified to double-stranded cDNA and then ligated with an adaptor. The amplified cDNA was purified using the magnetic bead-based method and the molar concentration was determined for each cDNA library. The HiSeq X Ten platform was used to perform transcriptome sequencing. Sequencing and subsequent bioinformatics analysis were completed at Novel Bioinformatics Co., Ltd (Shanghai, China).

### Quantitative RT-PCR

qRT-PCR was performed as previously described (16). Differentially expressed genes (DEGs) were verified by qRT-PCR using different samples like that in RNA-seq. Samples were amplified in a 20-μl reaction containing 10-μl 2 × SYBR® Premix Ex TaqTMII (TAKARA), 100 nM forward and reverse primers and 1-μl 10-fold diluted cDNA sample. 16S rRNA, which is constantly transcribed in bacteria, was chosen as the housekeeping gene. For each gene, qRT-PCR was performed in triplicate and relative transcription levels were determined using the 2^−ΔΔCt^ method. Primers used for qRT-PCR are provided in Supplementary Table 1.

### Cell Infections

The multiplication of *B. canis* RM6/66 and its derived strains in RAW264.7 macrophages were evaluated to determine the ability of Δ*mucR* mutant strain to survive intracellularly. The assays were performed as previously described (14). Briefly, in a permissive culture condition, *B. canis* strains were cultured in a flask with 0.2 μM vent cap for 12 h. RAW264.7 cells were seeded in 24-well plates (Corning, NY, USA) and then infected with *Brucella* strains at a multiplicity of infection (MOI) of 100. After 1 h of incubation, cells were washed thrice with phosphate buffer saline (PBS) and incubated in Dulbecco's Modified Eagle Medium (DMEM) containing gentamycin (50 μg/ml) to eliminate extracellular bacteria. At 1, 24, and 48 h post-infection (hpi), cells were washed and lysed with 500 μl 0.1% (v/v) Triton X-100 water solution, and then the bacteria count was determined by plating on TSA. The assays were run in triplicate and repeated at least twice.

### Mouse Infections

Virulence assay was performed using BALB/c mice as previously reported (14) with some modifications. Mice were inoculated intraperitoneally with 0.1 ml (1 ×10^7^ CFU) of *B. canis* RM6/66 and its derived strains. On the 7th and 28th day post-infection (dpi), mice were euthanized *via* asphyxiation (*n* = 4 per group), after which the spleens were removed, weighed, and homogenized in 1 ml of PBS. The bacterial colonies were counted by the TSA plate method and CFUs per spleen were calculated.

### Stress Assays

To test the susceptibility of *Brucella* strains under different stress conditions, modified stress assays were performed as previously reported (9, 15). *Brucella* strains (with an initial density of 1 ×10^6^ CFU/mL) were, respectively, cultured in an acidic tryptic soy broth (TSB) medium (pH 4.5) for 2 h. Afterward, the concentration of bacteria was measured by plate count and the survival rate (%) calculated as surviving bacteria relative to the TSB control.

To determine the sensitivity of Δ*mucR* mutant to heat shock stress and hypertonic environments, 5 μl gradient dilution of bacterial cultures (1 ×10^9^ CFU/mL) were cultured on TSA medium at 42°C or TSA medium containing 200 mM sodium chloride (NaCl) at 37°C for 72 h, respectively.

To compare the growth of *B. canis* RM6/66, Δ*mucR* and the complemented strain in low iron medium, the TSB medium was limited in iron by adding different concentrations of the iron chelator, 2,2-dipyridyl (2.5, 5, and 10 mM). The bacteria were cultured in this medium at the same initial density (1 ×10^6^ CFU/ml) and the CFUs were determined at 48 h for each strain.

### Determination of MICs of Antibiotics

MIC values of the antibiotics from both β-lactam (Ampicillin, Amoxycillin, and Carbenicillin) and non-β-lactam (Ciprofloxacin, Gentamicin, and Tetracycline) were determined as previously reported (16).

### Statistical Analysis

Differences between the means of the experimental and the control groups were analyzed using the independent-samples *t*-test using SPSS 17.0 program. The differences were considered to be significant at *p* < 0.05.

## Results

### Construction of Δ*mucR* Mutant Strain and the Complementary Strain C*mucR*

**Figure 1:**
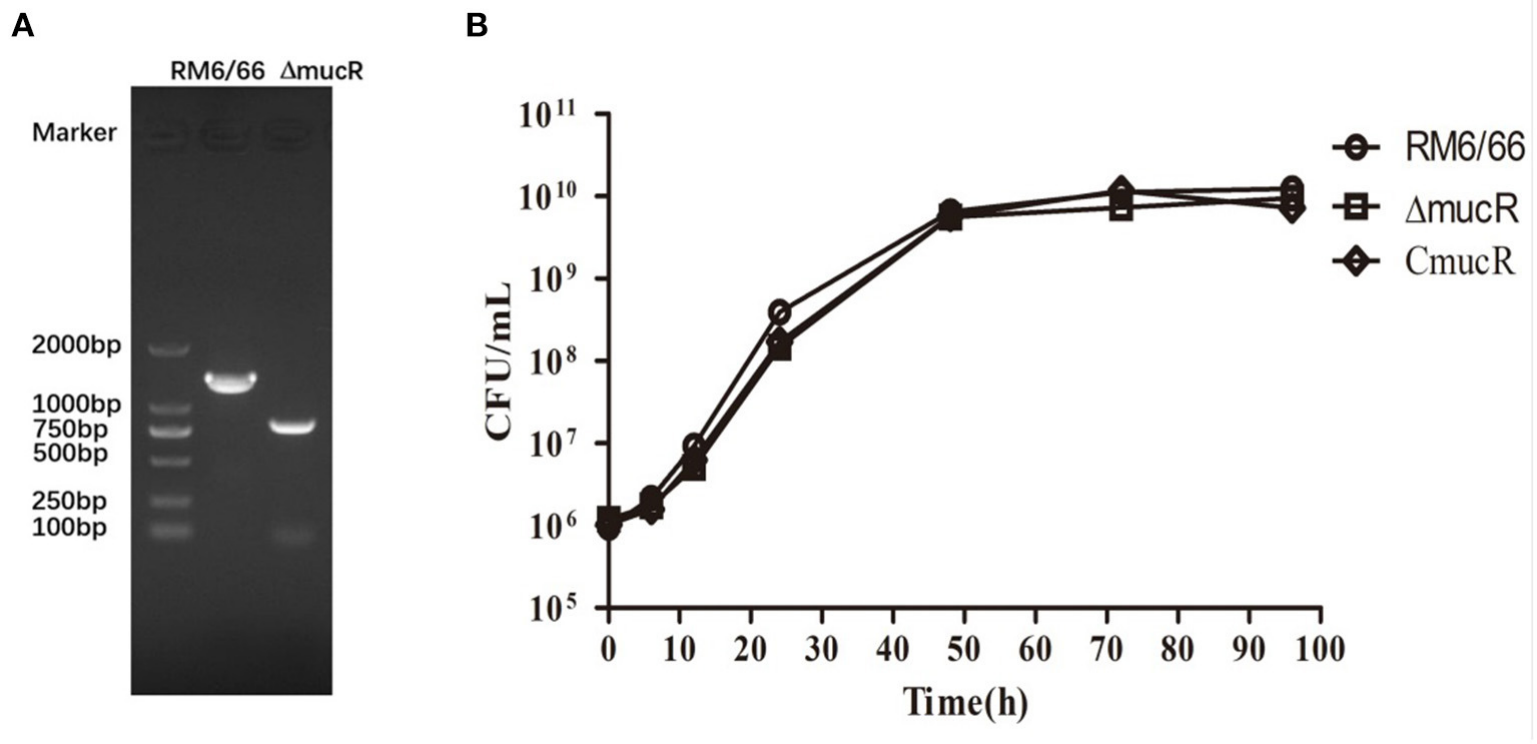
Characterization of *mucR* in-frame deleted mutant strain Δ*mucR*. **(A)** PCR amplification confirmed deletion of the *mucR* gene in Δ*mucR*. Lane M: DNA marker DL2000 (Takara, Dalian, China). *B. canis* RM6/66: a 1429-bp fragment was amplified using primer pair D-mucR-UF/D-mucR-DR. Δ*mucR*: an 846-bp fragment was amplified using primer pair D-mucR-UF/D-mucR-DR. Primer sequences are provided in Supplementary Table 1. **(B)** Deletion of *mucR* in *B. canis* RM6/66 did not affect bacterial growth. *Brucella* strains (initial density of 1 ×10^6^ CFU/mL) were grown in TSB at 37°C with continuous shaking for 96 h. The CFUs of *B. canis* RM6/66 and Δ*mucR* were measured at different time intervals. Statistical significance was determined using the unpaired Student's *t*-test. ns, no significance.

*B. canis* RM6/66 *mucR* deletion mutant was constructed *via* a double recombination event and confirmed by PCR (Figure 1A). *mucR* complement strain C*mucR* was constructed by restoring the *mucR* gene using the broad-range plasmid pMR11. The resultant strains were verified by PCR and sequencing.

### Deletion of *mucR* Showed No Difference in Growth

To characterize the cell growth rate of Δ*mucR* strain, the bacterial strains were analyzed in TSB medium at 37°C with the same initial density of 1 ×10^6^ CFU/ml. The result showed that the growth rate of Δ*mucR* was almost the same as its parent strain RM6/66 and the complementation strain C*mucR* at different time points (Figure 1B).

### Loss of MucR Impairs Survival of *B. canis* in Macrophages and Alters Virulence in BALB/c Mice

**Figure 2:**
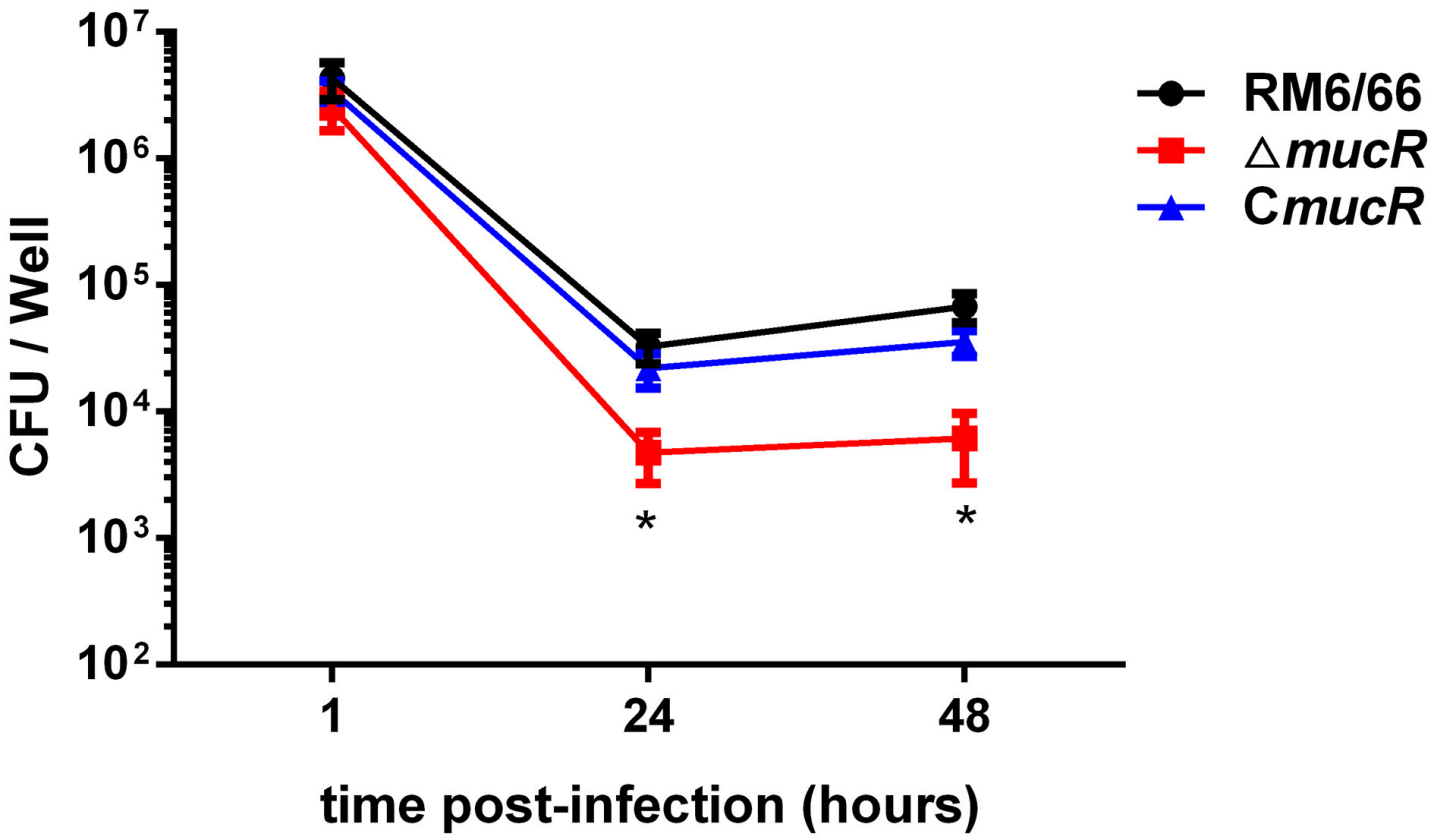
Role of mucR in the intracellular behavior of *B. canis* RM6/66 in RAW264.7 macrophages. Intracellular survival of *B. canis* RM6/66, Δ*mucR*, and C*mucR* strains in RAW264.7 macrophages. The MOI was 100:1. Data represent the means of three experiments performed in duplicate and error bars indicate the SD. *p < 0.05.

To assess the virulence of Δ*mucR* strain, the ability of Δ*mucR* to multiply within the cultured macrophage RAW264.7 was tested. As shown in Figure 2, the intracellular bacterial load of Δ*mucR* strain was almost the same as that of RM6/66 and C*mucR* at 1 h post-infection, while the bacterial load of Δ*mucR* was significantly reduced at 24 and 48 h (both with *p* < 0.05) in murine macrophage compared to RM6/66 and C*mucR* strains.

**Figure 3:**
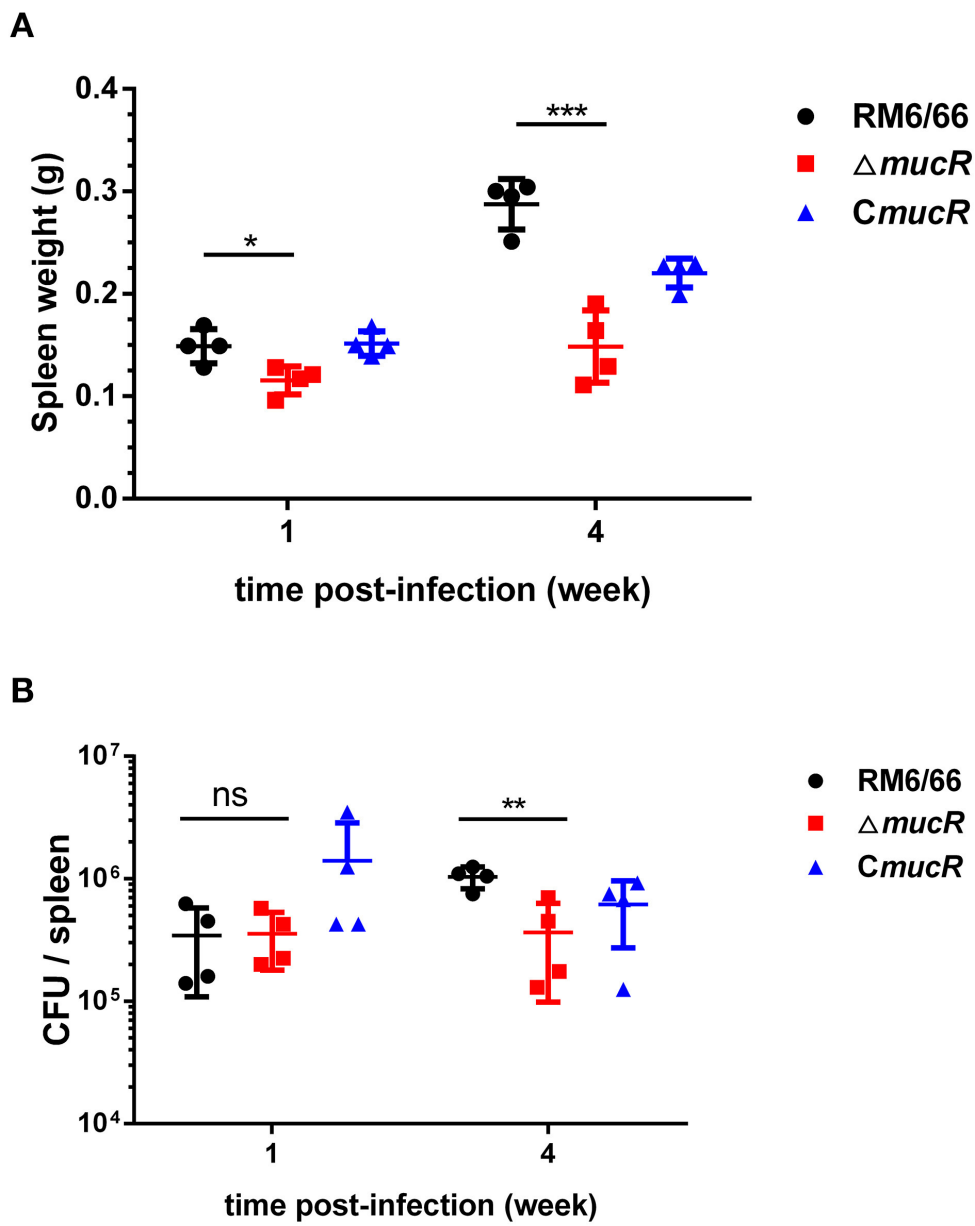
Evaluation of the residual virulence of *B. canis* RM6/66, Δ*mucR* and C*mucR* in BALB/c mice model. **(A)** Spleen weight of *B. canis* RM6/66, Δ*mucR*, and C*mucR* infected mice at 1 and 4 weeks post-infection. **(B)** Splenic CFUs of *B. canis* RM6/66, Δ*mucR*, and C*mucR* infected mice at 1 and 4 weeks post-infection. *p < 0.05, **p < 0.01, ***p < 0.001.

To assess the virulence of Δ*mucR* strain, the behaviors of Δ*mucR* and RM6/66 strains in an infected mouse model at 1 and 4 weeks were also analyzed. The spleen weight of the mice infected by the Δ*mucR* mutant infection group was significantly lighter than that of the RM6/66 infection group at both 1 (*p* < 0.05) and 4 (*p* < 0.001) weeks post-infection (Figure 3A). In addition, the CFU of Δ*mucR* mutant recovered from mice spleens was almost the same as that of RM6/66 at 1-week post-infection. However, the CFU number of *mucR* mutant recovered from the mice spleens was significantly lower at 4 weeks post-infection (*p* < 0.01) (Figure 3B).

### *mucR* Deletion Strain Was Sensitive to Heat Stress and Iron Limitation

**Figure 4:**
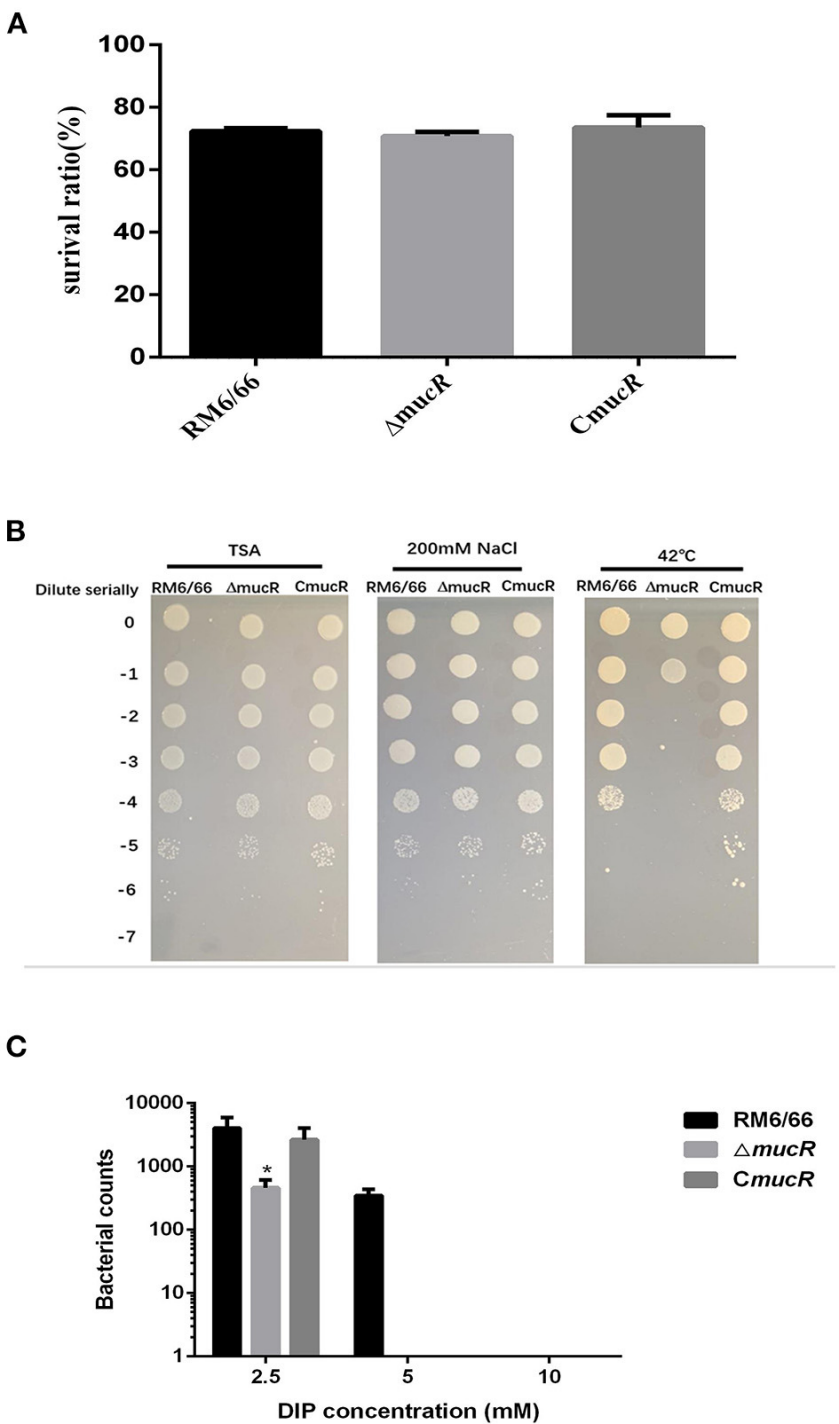
Growth behavior of *B. canis* RM6/66, Δ*mucR*, and C*mucR* strains under various stress conditions. **(A)** *Brucella* strains (initial density of 1 ×10^6^ CFU/ml) were grown in TSB (pH 4.5) at 37°C for 2 h. Viable counts were taken 2 h after the challenge. Values represent the mean from three independent experiments performed in duplicate. **(B)** 5-μl gradient dilution of bacterial cultures were, respectively, cultured on TSA medium at 42°C or TSA medium containing 200 mM NaCl at 37°C for 72 h. These experiments were independently repeated thrice. **(C)** Survival of *B. canis* RM6/66, Δ*mucR*, and C*mucR* strains in TSB medium containing various concentrations of 2,2-dipyridyl (iron chelator) was determined. The graph represents the average data from at least three independent experiments. Statistical analysis was performed with a *t*-test.

As shown in Figure 4A, the survival ratio of Δ*mucR* strain was not significantly affected in acidic TSB medium compared with RM6/66 strain and the complemented strain C*mucR* (Figure 4A). When exposed to a hypertonic environment, the survival ratio of Δ*mucR* was similar to that of the parental strain RM6/66 and C*mucR* (Figure 4B). However, when Δ*mucR* strain was cultured at 42°C for 72 h, the survival ratio of the mutant strain was significantly reduced than that of RM6/66 and C*mucR* (Figure 4B).

The growth patterns of Δ*mucR, B. canis* RM6/66, and the complemented strain in the low iron medium were also measured. In the concentrations evaluated, the presence of 2,2-dipyridyl restricted the growth of all three strains. As compared with *B. canis* RM6/66, the growth of Δ*mucR* was significantly reduced at 2.5 mM 2,2-dipyridyl (*p* < 0.05) and abolished at a higher concentration (Figure 4C).

### *mucR* Mutant Is More Sensitive to Various Antibiotics

The susceptibilities of RM6/66, Δ*mucR*, and C*mucR* to β-lactam and non-β-lactam drugs were examined using standard procedures. The sensitivity of *mucR* mutant to Ciprofloxacin, Gentamicin, and Tetracycline was similar to that of the parental strain RM6/66 or C*mucR*. However, this mutant showed a higher sensitivity to all the three types of β-lactam agents tested. Such enhancement in susceptibility, estimated by MICs, was two-fold compared with its parent strain (Table 1).

**Table 1:** MIC of β-lactam and non β-lactam antimicrobials for *B. canis* RM6/66 and mutants.

**Drug**	**MIC (μg/ml) for** ***B. canis***		
	**RM6/66**	**Δ*mucR***	**C*mucR***
**β-lactam antibiotics**			
Ampicillin	4	2	4
Amoxycillin	0.5	0.25	0.5
Carbenicillin	16	8	16
**Non** **β-lactam antibiotics**			
Tetracycline	0.25	0.25	0.25
Ciprofloxacin	0.25	0.25	0.25
Gentamicin	0.5	0.5	1

### MucR Affects the Expression of Flagellar Genes in *B. canis*

Previous studies on the role of MucR in *B*. *melitensis* 16M demonstrated that MucR protein could repress the expression of several flagellar genes (11). Thus, the expression of 15 flagellar genes in the *mucR* mutant strain was analyzed using qRT-PCR. Our results showed that the expression level of eight flagellar genes (*ftcR, fliC, flgC, flhA, flgB, fliP, flgK*, and *fliF*) was significantly increased in *mucR* mutant compared with its parent strain *B. canis* RM6/66 (Table 2).

**Table 2:** MucR affected the transcription level of the flagellar genes.

**Gene**	**Locus tag**	**Log_**2**_FC (Δ*mucR* vs. RM6/66)**
*ftcR*	DK60_2824	8.96
*fliC*	DK60_2831	4.91
*fliL*	DK60_3013	−0.94
*flgA*	DK60_3033	−0.46
*flgC*	DK60_3030	2.86
*flgE*	DK60_2823	−1.34
*fliN*	DK60_3007	0.9
*flhA*	DK60_2816	3.93
*flgB*	DK60_3029	3.65
*fliP*	DK60_3038	1.23
*fliQ*	DK60_2817	0.17
*fliR*	DK60_2815	0.30
*flgK*	DK60_2822	4.90
*fliF*	DK60_2830	5.08
*flgG*	DK60_3032	−0.83

### Identification of DEGs in *mucR* Mutant Strain

To uncover the underlying mechanism of virulence attenuation of Δ*mucR* strain, changes of the transcriptional profile of gene expression of Δ*mucR* or RM6/66 were analyzed using transcriptomic analysis. The transcriptional data set was submitted to the Gene Expression Omnibus (GEO) repository (National Center for Biotechnology Information, NCBI) with access number GSE155748. To confirm the RNA-seq data, 12 genes were chosen for qRT-PCR. Transcriptional data of all selected genes gave identical tendencies by both methods RNA-seq and qRT-PCR (Table 3).

**Table 3:** Transcriptional level of 12 randomly selected genes by RNA-seq and RT-qPCR.

**Locus tag**	**Log**_****2****_**FC (Δ*****mucR*** **vs. RM6/66)**		**Function**
	**qPCR**	**RNA-seq**	
DK60_1789	−1.167	−2.025	ATP synthase F1, epsilon subunit
DK60_1816	−1.777	−2.092	Signal recognition particle protein
DK60_2150	−2.397	−2.923	Opacity porin family protein
DK60_3002	1.47	1.173	HTH-type quorum sensing-dependent transcriptional regulator vjbR
DK60_2832	7.563	7.421	Autotransporter-associated beta strand repeat family protein
DK60_2794	4.103	6.656	PemK-like family protein
DK60_291	4.393	6.577	Bacterial regulatory s, luxR family protein
DK60_2276	−2.517	−2.165	Bacterioferritin
DK60_1531	2.933	7.728	Bacterial SH3 domain protein
DK60_2952	0.612	0.889	P-type DNA transfer protein VirB5
DK60_2949	−0.49	−0.077	Type IV secretion system protein virB8
DK60_647	−6.287	−8.581	Transcriptional regulatory protein ros
DK60_1976	6.183	7.005	Aquaporin Z 2

**Figure 5:**
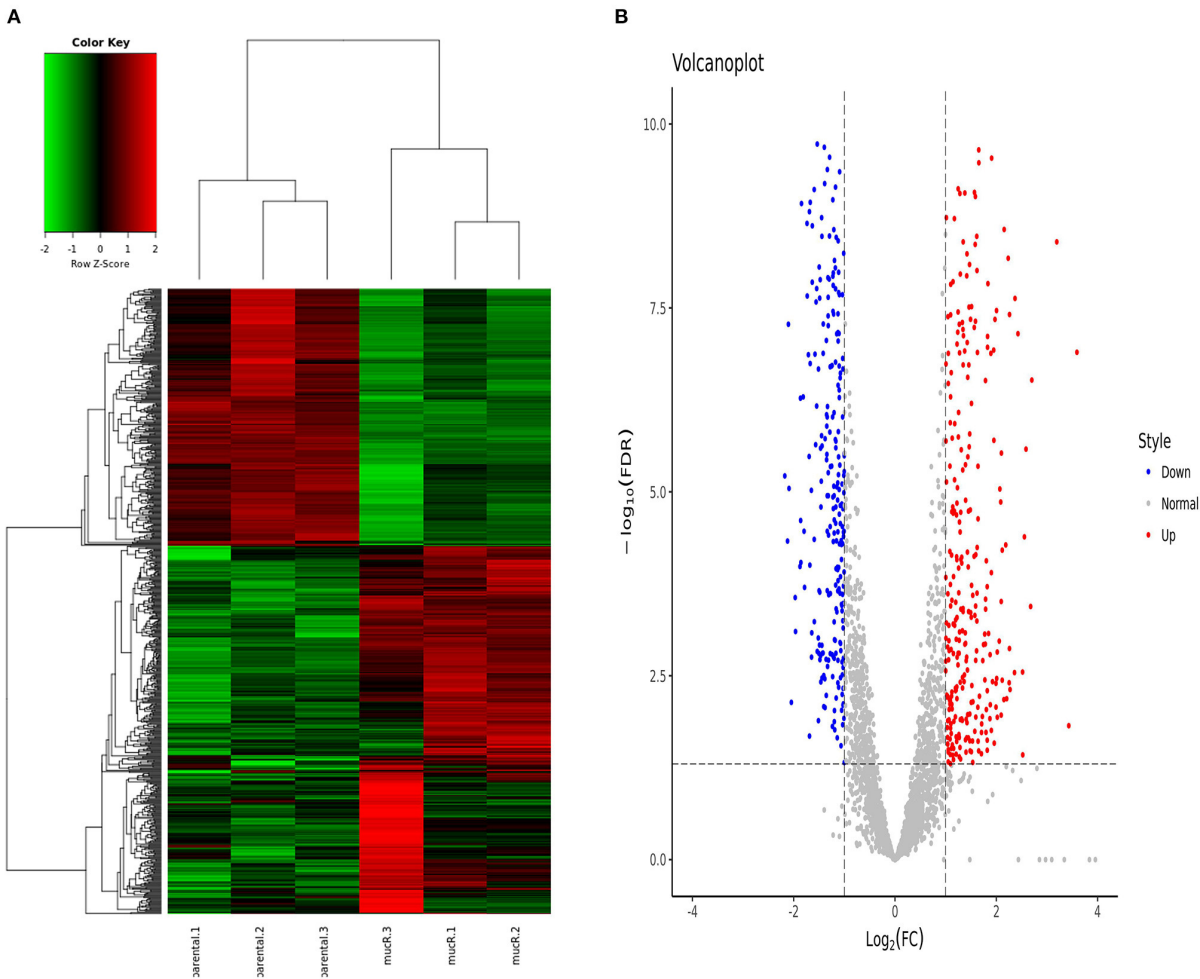
Differentially expressed genes identified by RNA-seq (*n* = 3 per group). **(A)** Heat map of the comparative transcriptome analysis of *B. canis* RM6/66 and Δ*mucR*. **(B)** Volcano plot of relative RNA expression from Δ*mucR* as compared to *B. canis* RM6/66. Genes in the upper left and right quadrants are significantly differentially expressed.

In total, 694 genes exhibited significant difference (log_2_ FC > 1 or < -1 and FDR <0.05). Among these genes, 409 genes were significantly up-regulated and 285 genes were significantly down-regulated in Δ*mucR* compared to its parent strain RM6/66 (Figures 5A,B). Genes, such as DK60_1986, DK60_2665, DK60_1987, K60_1068, DK60_1531, DK60_1985, DK60_2016, DK60_2832, DK60_1984, and *aqpZ*2, were among the top 10 up-regulated genes, while DK60_2150, DK60_1727, DK60_895, *bfr*, DK60_3051, DK60_2926, *ffh*, DK60_1851, *atpC*, and DK60_1852 were among the top 10 down-regulated genes.

**Figure 6:**
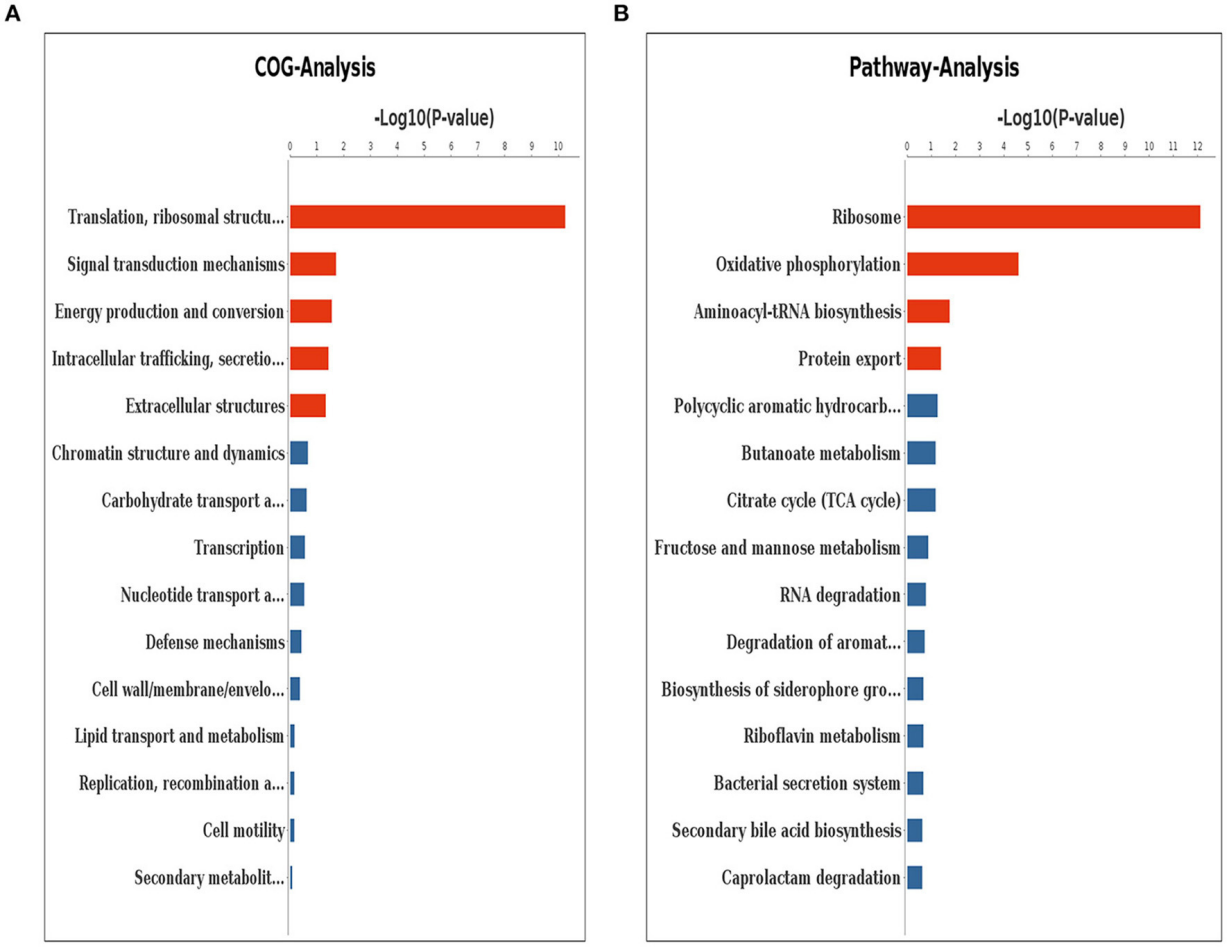
Clusters of orthologous genes (COG) analysis **(A)** and KEGG pathway analysis **(B)** for differentially expressed genes. The red columns indicate that these categories or pathways were significantly enriched (*p* < 0.05), while the blue columns imply that these categories or pathways were not significantly enriched.

According to the result of clusters of orthologous genes (COG) analysis, DEGs were mainly involved in translation, ribosomal structure and biogenesis, signal transduction mechanisms, energy production and conversion, intracellular trafficking, secretion and vesicular transport, and extracellular structures (Figure 6A). The Kyoto encyclopedia of genes and genomes (KEGG) pathway analysis demonstrated that the genes which exhibited significant difference were primarily enriched in the ribosome, oxidative phosphorylation, aminoacyl-tRNA biosynthesis, and protein export (Figure 6B).

## Discussion

In the present study, we report extensive characterization of an in-frame deletion mutant of *B. canis mucR* for the first time. This gene was previously found to regulate virulence in both cellular and murine models of infection in *B. melitensis* and *B. abortus* (11, 13). According to the result of the virulence assay, it was found that the *mucR* gene was also crucial for *B. canis* virulence in both the macrophage infection model and the murine infection model.

In previous studies, deletion of *mucR* gene in *B. abortus* 2308 and *B. melitensis* 16M resulted in a significant growth deficiency and mutant strains produced smaller-size colonies when grown on a solid medium (11, 13). However, we found that the growth curve of RM6/66, Δ*mucR*, and *CmucR* was almost the same when cultured in a TSB medium with the same initial concentration. Moreover, the sizes of the three *B*. *canis* colonies on blood agar had no significant difference.

In smooth-type *Brucella* strain, MucR protein contributed to the development of resistance to various stress conditions, such as acidic pH, iron-limitation, oxidative stress, cationic peptide, and detergents (9, 11, 13). Thus, we also comprehensively detected the stress response of *mucR* mutant strain in different conditions in this study. Our results indicated that the *mucR* mutant was more sensitive to heat stress and iron deficiency. In addition, reduced tolerance to these stress conditions, which might be encountered in the host macrophage, could be possible reasons for the attenuation of the *mucR* mutant strain.

In *B. melitensis*, MucR protein was found to be a repressor of flagellar gene expression (11). Based on the RNA-seq data, the expression of four flagellar genes was significantly up-regulated and the result of qRT-PCR also indicated that MucR could repress the transcription of flagellar genes in *B. canis*. Besides, it was reported that the expression of flagellar genes was tightly regulated by the QS regulator VjbR, RpoE1, and RpoH2 in *B. melitensis* (17–19). A recent study from our lab indicated that deletion of the QS regulator VjbR also affected the transcription level of the *ftcR* gene in *B. canis* (14). Whether, flagellar genes of *B. canis* were also regulated by RpoE1 or RpoH2 should be further explored.

To further uncover the mechanisms underlying virulence attenuation in the *mucR* mutant strain, we characterized the transcriptional profile of RM6/66 and Δ*mucR* by transcriptome analysis and up to 694 genes were found to be differentially expressed over two-fold in the mutant strain. The number of up-regulated genes was more than that of the down-regulated genes, which was consistent with the RNA-seq data in *B. melitensis mucR* strain (9). It seemed that MucR protein should be a global negative regulator in *Brucella* strains.

Iron is a necessary element for brucellosis survival (20). Bfr protein played an important role in controlling iron homeostasis, which accounted for about 70% of the intracellular iron content in *B*. *abortus* (21). Moreover, the heme transporter BhuA was reported to be related to *Brucella* virulence both *in vitro* and *in vivo* (22). In our study, we found that *mucR* mutant was more sensitive to iron-limitation stress and the result of RNA-seq in our study also showed that the expression of *bfr, bhuA*, and DK60_1666 (encoding ferric uptake regulator family protein) was affected. Thus, we speculated that the change in iron-limitation tolerance could also be a result of the abnormal expression of these iron metabolism-related genes.

During bacterial infection pathogen-host interactions expose bacteria to various physiological and biological stresses, including heat stress. To avoid degradation by the host cell defense systems, intracellular pathogens could adapt to changes in their environment by coordinated of gene expression (23). In previous study, the protein synthesis pattern in response to heat stresses were studied by two-dimensional polyacrylamide gel electrophoresis, and the expression of several proteins were increased, such as GroEL, DnaK, AapJ, and Fe and/or Mn SOD (24). In our study, the result of RNA-seq showed that the expression of *aapJ, groEL*, and DK60_400 (encoding hsp33 family protein) was affected. It seemed that the change in heat stress tolerance might be a result of the abnormal expression of these genes mentioned above.

Beta-lactam antibiotics can interfere with the final stage of cell wall synthesis by modifying activities of enzymes and penicillin-binding proteins (25). Based on RNA-seq results, up to 38 genes involved in cell wall/membrane/envelope biogenesis (COG: M) were found to be differently expressed in *mucR* deletion strain. Thus, it seemed that the reduced tolerance to β-lactam antibiotics in the *mucR* mutant should be due to changes in bacterial surface integrity.

## Conclusion

The current study indicated that the transcriptional regulator MucR was essential for bacterial resistance to heat stress, iron limitation and antibiotics, as well as bacterial virulence in *B. canis*. In addition, various genes linked to the phenotype changes of *mucR* mutant were uncovered using RNA-seq assay. Our study not only reveals the pathogenic mechanism of *B. canis* but also provided insight into MucR regulon in different *Brucella* strains.

## Data Availability Statement

The datasets presented in this study can be found in online repositories. The names of the repository/repositories and accession number(s) can be found below: https://www.ncbi.nlm.nih.gov/, GSE155748.

## Ethics Statement

The animal study was reviewed and approved by the Animal Ethics Committee of China Institute of Veterinary Drug Control. The affiliation of this ethics committee was NO.33 Qingfeng Road, Daxing District, Beijing.

## Author Contributions

HD, YQ, and JD designed the experiments. JS, XP, YL, HJ, YF, and QL performed the experiments. JS and HD prepared the manuscript. YL and LZ analyzed the data. All authors have read and approved the final version of this manuscript.

## Conflict of Interest

The authors declare that the research was conducted in the absence of any commercial or financial relationships that could be construed as a potential conflict of interest.
