# Supplementary material for: Deletion of the Transcriptional Regulator MucR in Brucella canis Affects Stress Responses and Bacterial Virulence
Source: Front Vet Sci. 2021 Jun 25;8:650942. doi: 10.3389/fvets.2021.650942 (PMC8267065; doi:10.3389/fvets.2021.650942)
Supplement: Supplementary file 1 [file Table_1.doc]

Table S1 Strains, plasmids and primers used in this study

| Plasmid or Primers | Description | Source or reference |
| --- | --- | --- |
| **Bacterial strains** |  |  |
| *B. canis* RM6/66 | *Brucella canis* wild type strain | CVCC70701 |
| Δ*mucR* | *Brucella canis* RM6/66 *mucR* gene deletion mutant strain | This study |
| C*mucR* | Amp^R^; Δ*mucR* containing *mucR* gene complementary plasmid | This study |
| C*mucR*1 | Cm^R^; Δ*mucR* containing *mucR* gene complementary plasmid | This study |
| *E. coli* (DH5α) | F^−^ φ80*lacZ*∆M15∆(*lacZ*YA-*arg*F)U169 *rec*A1 *end*A1 *hsd*R17(r_k_^−^, m_k_^+^) *pho*A *sup*E44 *thi*-1 *gyr*A96 *rel*A1 λ^−^ | Tiangen |
| **Plasimids** |  |  |
| puc19-*sacB* | Amp^R^; pUC19 plasmid containing *SacB* gene | This study |
| PBBR1MCS-1 | Cm^R^; broad host range cloning vector | Kovach *et al*. 1994 |
| **Pirmers** |  |  |
| D-MucR-UF | GGTACCTCCGATGCGAGTTGTTT (*kpn*I underlined) | This study |
| D-MucR-UR | CAATGGCAACCCACCCCTT | This study |
| D-MucR-DF | TTGCCATTGCGGTTCCAACGACT | This study |
| D-MucR-DR | CCCAAGCTTAAATCGACAGGAATGC (*Hind*Ⅲ underlined) | This study |
| C-MucR-F | GGGATCCATCTCTATATAGTTGCCGG (*Bam*HI underlined) | This study |
| C-MucR-R | GGGTACCAAATCGACAGGAATGCA (*kpn*I underlined) | This study |
| 16S rRNAf | TACCAGCCCTTGACATCC | This study |
| 16S rRNAr | TCATCCCCACCTTCCTCT | This study |
| qAqpZ2-F | GGTCTCGCCTCGAACGGTTAT | This study |
| qAqpZ2-R | GAGCCCAGAATGATGATGATGAAG | This study |
| qAtpC-F | CCGCAACGCATGTGGACGATA | This study |
| qAtpC-R | TTTCCGCCTTGGTGCGATGTT | This study |
| qBfr-F | AGCTGATTGATCGCATTATCTTCC | This study |
| qBfr-R | CGAGCGTCATATTCACCCTTGA | This study |
| qVjbR-F | TTCTTATGACGCTGCCTGCTA | This study |
| qVjbR-R | GGGCAAATATCCGCATCTTCT | This study |
| qDK60_1531-F | GGGTGGCAGGCCCAACATCTA | This study |
| qDK60_1531-R | TGACCAAAGCCGTGACCGAAA | This study |
| qDK60_2150-F | AGCGTTACGGGTCCGATTTCA | This study |
| qDK60_2150-R | TCGGTAGCCGTGTAGCCAAGA | This study |
| qDK60_2794-F | ACGGGCTCGATAACCATTTGC | This study |
| qDK60_2794-R | GGTCGTGATCTTGTCCACCATAA | This study |
| qDK60_2832-F | TGGCTATGACATCACCGTTTCC | This study |
| qDK60_2832-R | CGATTGTGGTCTTGCCCGTAT | This study |
| qFfh-F | GCGCTGATCGAGGCGGATGTT | This study |
| qFfh-R | TCTGGCCGGGCTTGATGCTTT | This study |
| qRos-F | GCCAGCGGTGAACCCGAAGAA | This study |
| qRos-R | TGCTCAGGCGTCATGTTGTAAT | This study |
| qBabR-F | TCGGCCTCTTGAGCTTTGTAC | This study |
| qbabR-R | TGGATAGATGCAGGAGATTGGT | This study |
| qVirB5-F | TCTACGAAGCGGTGATGAG | This study |
| qVirB5-R | TTGTCGGAGATGGAGGC | This study |
| qVirB8-F | AGCGTTGAACTGGGAGG | This study |
| qVirB8-R | CCGAGCAGCACGGTAAT | This study |
| qFlgA-F | GGCTCTTGCTGGTGGCTTTC | This study |
| qFlgA-R | GCGGTGTACCCATCGCAGTTAT | This study |
| qFlgB-F | CGCAGTGGTTGTCGGTGAGA | This study |
| qFlgB-R | CGCCTTGACGATGCTGGTGTT | This study |
| qFlgC-F | GAGGTTCGGGTTGCGGAAGT | This study |
| qFlgC-R | TTATGTGCTCCTCAAGAGGTCGA | This study |
| qFlgE-F | GGCTCCACCTATCTGACACGG | This study |
| qFlgE-R | CGGTTCGGGCTTGCTACATT | This study |
| qFlgG-F | CAGCTTGTGACGCTTGACGG | This study |
| qFlgG-R | CGGCCTGAATAACCTTGGAGTT | This study |
| qFlgK-F | GCGATAGCGTGGTCTCGGTTGC | This study |
| qFlgK-R | CCTTGCGTGCCGTCGTCTCA | This study |
| qFlhA-F | GGTGAGGAAGTGCGTGAGCC | This study |
| qFlhA-R | GTGCCGAACTGTTCCAGAAGG | This study |
| qFlic-F | TCTCAGCAATGCCAACTACGC | This study |
| qFlic-R | CGTGCCGATTCCTTGTTCAT | This study |
| qFlif-F | GCCCATCCTACGAGACGCTCTA | This study |
| qFlif-R | CACATGGCTCGCAACCTGCT | This study |
| qFliI-F | CGCCTGATCTGCCACTTACCC | This study |
| qFliI-R | AGCGTTGATTTGCCGACACC | This study |
| qFliN-F | CGGTGGATGTGCAGGTGGTT | This study |
| qFliN-R | TGCCGATGATTTCGGTGAGG | This study |
| qFlip-F | GAAGCACTCCCTCGGTCTCG | This study |
| qFlip-R | GCAATGGAAAGCACGGTCAA | This study |
| qFliQ-F | TCGGCGATCTGGACTGTGCT | This study |
| qFliQ-R | GGTCATTTCCTGAATTTGCGTCA | This study |
| qFliR-F | GCCTGATGGTGGGTGAAATG | This study |
| qFliR-R | CAGAATGGCAAAGACGATAAAGG | This study |
| qFtcR-F | GAAGCCTTCCTGATTGGTGAA | This study |
| qFtcR-R | AATGCCGTAAATCGCACTAAAA | This study |

^R^ Antibiotic resistance.
